# Progress of Thomsonism

**Published:** 1844-06

**Authors:** 


*Progress of Thomsonism.*—On the 29th page of the Botanico-Medical Recorder, is a communication from one H. Sheddon, dated at Coventry, R. I., which is entirely a unique production in several respects. He speaks of himself as being located at Coventry, as a "Thomsonian physician." And further, in speaking of the people of that quarter, he says, "they begin to think that when people die, it is not always the Lord's will, but the doctor's poison. These poisoners have done everything that is dishonorable, low and mean, to stop the progress of Thomsonism in Coventry." No wonder some have risen up in their strength to withstand the tidal wave of such concentrated ignorance and presumption as mark the character of this correspondent's production. But it is chiefly in his poetical light that this man of Coventry is to be noted. With a boldness that brooks no control, and a fervency such as poets feel in moments of ideal exaltation, Dr. Sheddon thus exclaims:

"But after all their foul abuse,
The people love Lobelia juice;
Steam and powders is the cry,
Let us have these, or we die."

He adds—"The first two years of my living in this place, I did not receive one hundred dollars." The wonder to us is, how he ever received anything.

*Boston Med. and Surg. Jour.* for May.

